# Auricular acupuncture for prehypertension and stage 1 hypertension: study protocol for a pilot multicentre randomised controlled trial

**DOI:** 10.1186/1745-6215-14-303

**Published:** 2013-09-22

**Authors:** Joo-Hee Kim, Hyun Jung Jung, Tae-Hun Kim, Seunghoon Lee, Jung-Eun Kim, Kyung-Won Kang, So-Young Jung, Ae-Ran Kim, Hyo-Ju Park, Mi-Suk Shin, Kyung-Min Shin, Hee-Jung Jung, Seung-Deok Lee, Kwon-Eui Hong, Sun-Mi Choi

**Affiliations:** 1 Acupuncture, Moxibustion & Meridian Research Group, Medical Research Division, Korea Institute of Oriental Medicine, Daejeon, South Korea; 2 Department of Acupuncture & Moxibustion, College of Korean Medicine, Kyung Hee University, Seoul, South Korea; 3 Department of Diagnostics, College of Oriental Medicine, Deagu Hanny University, Daegu-si, South Korea; 4 College of Korean Medicine, Gachon University, Seongnam, South Korea; 5 Department of Acupuncture & Moxibustion, College of Oriental Medicine, DongGuk University, Seoul, South Korea; 6 Department of Acupuncture & Moxibustion, College of Oriental Medicine, Dae-Jeon University, Daejeon, South Korea

**Keywords:** Hypertension, Auricular acupuncture, Effect, Safety, Clinical research protocol

## Abstract

**Background:**

Hypertension, a worldwide public health problem, is a major risk factor for cardiovascular and kidney disease, and the medical and economic burden of hypertension is increasing. Auricular acupuncture has been used to treat various diseases, including hypertension. Several studies have shown that auricular acupuncture treatment decreases blood pressure in patients with hypertension; however, the scientific evidence is still insufficient. Therefore, we aimed to perform a randomised controlled clinical trial in patients with prehypertension and stage 1 hypertension to evaluate the effect and safety of auricular acupuncture.

**Methods/designs:**

This on-going study is a two parallel arm, assessor-blinded, randomised controlled trial. Sixty participants with prehypertension and stage 1 hypertension will be recruited and randomly allocated into two groups in a 1:1 ratio. Participants in the auricular acupuncture group will receive auricular acupuncture treatment two times per week for 4 weeks. Participants in the usual care group will not receive any acupuncture treatment during the study period. All participants in both groups will be provided with verbal and written educational materials regarding the dietary and physical activity habits for controlling high blood pressure, and they will self-manage their lifestyle, including diet and exercise, during the study. The primary outcome is the 24-h average systolic and diastolic blood pressure, as measured with an ambulatory monitor. The secondary outcomes are the mean change in the average systolic and diastolic blood pressure during day- and night-time, the circadian rhythm of blood pressure, the mean arterial pressure, the change in blood pressure before and after auricular acupuncture treatment, the EuroQOL-5D (EQ-5D), heart rate variability (HRV), body mass index (BMI) and laboratory examination, including lipid profile and high sensitivity C-reactive protein (hs-CRP). Safety will be assessed at every visit.

**Discussion:**

This pilot multicentre randomised controlled trial will explore the feasibility of further auricular acupuncture research and provide important clinical evidence for the effect and safety of auricular acupuncture on blood pressure in patients with prehypertension and stage 1 hypertension compared with usual care.

**Trial registration:**

Clinical Research Information Service: KCT0000169

## Background

Hypertension, a worldwide public health problem, is a major risk factor for cardiovascular and kidney disease. The prevalence and global burden of the disease are increasing [1]. Stage 1 hypertension is systolic blood pressure (SBP) varying from 140 to 159 mmHg or diastolic blood pressure (DBP) varying from 90 to 99 mmHg, and it is a common form of hypertension. The Seventh Report of the Joint National Committee on Prevention, Detection, Evaluation and Treatment of High Blood Pressure (JNC7) defined prehypertension as SBP of 120 to 139 mmHg and DBP of 80 to 89 mmHg to identify individuals who were associated with a risk of developing hypertension and decrease blood pressure (BP), inhibit the progression of BP to hypertensive levels with age and prevent hypertension through early intervention [2].

The current treatments for hypertension include several classes of antihypertensive drugs and strategies to modify the effect of adverse lifestyles on blood pressure. Non-pharmacological interventions, including weight loss, reduced sodium intake, regular physical activity, smoking cessation, and the moderation of alcohol intake, are recommended [2,3]. Antihypertensive drugs, including angiotensin-converting enzyme (ACE) inhibitors, angiotensin receptor blockers (ARBs), beta-blockers, calcium channel blockers (CCBs), and thiazide-type diuretics, are commonly used [2]. However, it is still controversial whether antihypertensive drug therapy is needed for persons with prehypertension [4,5] and mild hypertension [6,7]. Long-term compliance with lifestyle modifications is also difficult for the patient.

Acupuncture has been widely used to treat various conditions, including cardiovascular disease and hypertension [8]. The mechanisms underlying the BP-lowering effect of acupuncture are associated with the control of BP modulators such as renin [9], aldosterone [10], angiotensin II [11] and endothelin-1 [12], and neurotransmitter modulation such as glutamate, GABA, serotonin and endocannabinoids [13]. Of three high-quality, randomised, controlled trials, two showed a positive effect on BP [14,15], but one trial reported a non-significant BP reduction between the groups [16]. Auricular acupuncture is one type of acupuncture based on the understanding that the external ear represents all parts of the human body, including the internal organs, and provides acupuncture points corresponding to these parts [17,18]. Several articles have evaluated the effect of auricular acupuncture on controlling BP [19-22]. Some studies also reported that the electroacupuncture treatment of auricular acupoints decreased BP in patients with hypertension [23,24]. However, the scientific evidence is insufficient [25]. Most previous studies have some methodological limitations, including low quality of the study design, small sample size, inadequate control groups, and a lack of suitable outcome measures. In addition, there is no clinical trial evaluating the effect of auricular acupuncture on prehypertension. Safe and effective interventions that are able to manage pre/mild hypertension would be desirable. Therefore, more rigorous studies are needed to elucidate the effect and safety of auricular acupuncture on pre/mild hypertension.

In the present study, we aimed to perform a randomised controlled clinical trial in patients with prehypertension and stage 1 hypertension to evaluate the effect and safety of auricular acupuncture compared with usual care. The 24-h ambulatory blood pressure will be used as the primary endpoint. The results derived from this study will also be used to explore the feasibility of auricular acupuncture treatment and calculate the appropriate sample size for a future large clinical trial.

## Methods/design

### Study design

**Figure 1:**
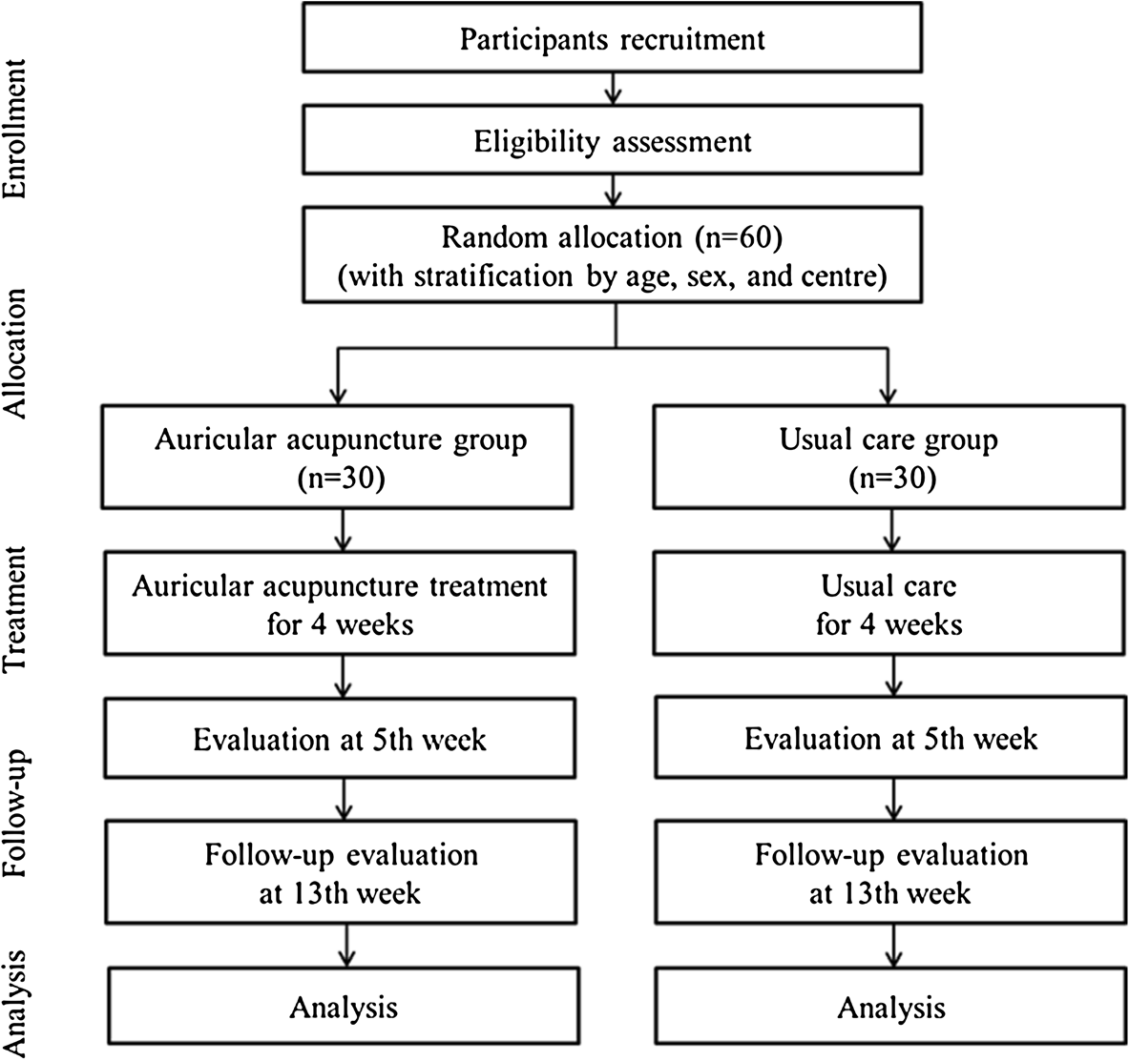
A flowchart of the study.

This study is a two parallel arm, assessor-blinded, randomised controlled trial. The trial will be performed at two clinical research centres in Korea in accordance with the Declaration of Helsinki and the Guidelines for Good Clinical Practice: the Korea Institute of Oriental Medicine (Daejeon University Hospital) and Dongguk University Ilsan Oriental Hospital. This protocol was registered with the ‘Clinical Research Information Service’, Republic of Korea, which is a registry in the WHO Registry Network. Eligible participants will be stratified by centre, age, and sex and randomly allocated into one of the two groups (the auricular acupuncture group or the usual care group) at a 1:1 allocation ratio and receive treatment for 4 weeks, with 2 months of follow-up (Figure 1). The evaluation of participants and the analysis of the results will be performed by professionals blinded to the group allocation.

### Participants

#### Inclusion criteria

A total of 60 patients will be recruited through local advertising and from the outpatients of the two hospitals. The eligibility criteria for the study include age 19 to 65 years; prehypertension (SBP of 120–139 mmHg or DBP of 80–89 mmHg) or stage 1 hypertension (SBP of 140–159 mmHg or DBP of 90–99 mmHg) according to JNC 7 criteria; and signed written informed consent. After providing consent, the blood pressure of potential subjects will be measured three times. Patients will be eligible for inclusion in the study if the average of three systolic or diastolic pressure measurements is within the specified intervals.

#### Exclusion criteria

Participants will be excluded if they are experiencing or have a history of the following: currently taking antihypertensive prescription or OTC medication or herb/supplements for controlling blood pressure; secondary hypertension; cerebrovascular or cardiovascular disease history; malignant cancer history; diabetes mellitus requiring medication or insulin injection; disease history, including renal diseases, hepatic diseases, thyroid diseases, active tuberculosis, and other infectious diseases; haemorrhagic diseases or taking anticoagulants; T-needle allergy; local infection at the ear; systemic steroid therapy or immune depressants; medications that may affect blood pressure, such as oral contraceptives, central nervous system depressants or stimulants; pregnant or breast-feeding women or women who plan to become pregnant; the use of traditional Korean medicinal treatment during the last 1 month; or inappropriate for this trial for other reasons, including unwillingness to comply with this study protocol or inability to complete the study-related questionnaires by themselves or with assistance, as decided by researchers.

### Randomisation

Study participants who meet the eligibility criteria will be randomly assigned to two groups (the auricular acupuncture group or the usual care group) at the second visit through central randomisation in a 1:1 ratio. Randomisation will be conducted using a computer-generated random allocation sequence through the stratified block randomisation method of the SAS package (version 9.1.3; SAS Institute, Inc., Cary, NC, USA) by a statistician with no clinical involvement in this trial, and the patients will be stratified by centre, age (19–30, 31–40, 41–50, 51–65 years) and sex.

Allocation concealment will be ensured, as the randomisation code will be released after the participants are recruited into the trial and all baseline measures are taken. The subjects and practitioners will be aware of the allocation arm according to the routine care setting. However, the outcome assessors and the statistician performing the data analyses will be masked to the treatment allocation [26].

### Intervention

#### Auricular acupuncture treatment group

Patients in the auricular acupuncture group will receive auricular acupuncture treatment twice a week for 4 weeks. Auricular acupuncture, which is attached to the skin, will be used for this trial, and Haeng Rim T-needles (Haeng Rim Seo Won Medical Co., Korea) will be adopted for intervention. The acupuncture points were selected by an expert committee composed of professors and researchers who specialise in traditional Korean medicine on the basis of a literature review [20,22] and a textbook [17] about acupuncture for blood pressure. At every visit, after the acupuncture points have been sterilised with 75% alcohol preparation pads, T-needles will be applied to six ear acupuncture points: the ear apex point (HX6, 7i), the endocrine point (CO 18), the *shen men* point (TF4), the superior triangular fossa point (TF1), the heart point (CO15), and the groove of the posterior surface point (PS). Attached T-needles should be maintained for 3–4 days and then removed. In addition, T-needles will be attached on one ear in the first session and the opposite ear in the next session, alternating thereafter.

The auricular acupuncture treatment will be conducted by doctors of Korean medicine who are certified by the Korean Ministry of Health and Welfare with at least 4 years of clinical experience and who have received more than 6 years of oriental medicine college education. They will take a 1-day training course for this trial, and the techniques for auricular acupuncture treatment will be standardised between practitioners. In addition, all study protocols and details, including the recording method for the clinical record form, outcome assessment methods, and monitoring process, will be standardised between the two centres through workshops before the beginning of the study. Additional treatment, including antihypertensive drugs and treatment with traditional Korean medicine, will not be allowed during the trial.

#### Usual care group

Subjects randomised to the usual care group will not be given auricular acupuncture treatment. However, participants in both groups will be provided with verbal and written educational materials regarding dietary and physical activity habits for controlling high blood pressure and self-manage their lifestyle, including diet and exercise, during the study. Additional treatment, including antihypertensive drugs and treatment with traditional Korean medicine, will be prohibited, and any violations will be recorded.

### Outcome

#### Primary outcome measurement

The primary outcome measure is the 24-h mean systolic and diastolic blood pressure. The subjects will undergo 24-h ambulatory blood pressure monitoring (PressureTrak Oscillometric Ambulatory Blood Pressure Monitor, SunTech, Morrisville, NC, USA), and the monitor will be programmed to record blood pressure measurements twice per hour during daytime (7 a.m. to 10 p.m.) and once per hour during night-time (10 p.m. to 7 a.m.). The mean systolic and diastolic blood pressure during the entire 24-h monitoring period will be used in the data analysis.

#### Secondary outcome measurements

We will assess the mean change in the mean systolic and diastolic blood pressure during daytime and night-time. The participants will be instructed to fill out a diary to record the time of sleeping and rising, and the readings will be divided into daytime and night-time values according to individual wake times and bedtimes. In addition to the mean systolic and diastolic blood pressure, the circadian rhythm of blood pressure and the mean arterial pressure will also be evaluated.

The change in blood pressure before and after auricular acupuncture treatment will be assessed. Blood pressure will be observed and recorded at every visit. At every visit, the brachial blood pressure will be measured three times in at least 1-min intervals using an automatic blood pressure monitor (FT-700 R; Jawon medical, Kungsan-city, Korea) with the subjects resting for at least 5 min in a quiet and temperature-controlled room with their upper arm at heart level. Subjects will be instructed not to drink caffeinated drinks such as coffee or tea and not to exercise, smoke, or eat 2 h before the blood pressure test [27]. The mean of these three measurements will be used in the data analysis.

The EuroQOL-5D (EQ-5D) will be used to evaluate health-related quality of life (HRQOL). The EQ-5D questionnaire is the most widely used instrument to measure health status, and it assesses scores across five dimensions of health: mobility, self-care, usual activities, pain or discomfort, and anxiety or depression. Each dimension is divided into three categories: no problems, some problems, and extreme problems. The Korean version of the EQ-5D has been developed, and its validity and reliability have been demonstrated [28].

Heart rate variability (HRV) is a useful non-invasive tool to investigate the sympathetic and parasympathetic function of the autonomic nervous system and cardiac autonomic dysregulation in hypertension. An SA-6000 (Medicore Co. Ltd., Seoul, Korea) will be used for studying HRV. The results, including the standard deviation of all of the normal-to-normal intervals (SDNN), the square root of the mean squared differences of successive normal-to-normal intervals (RMSSD), the percentage of successive normal interbeat intervals greater than 50 ms (pNN50), the very low frequency (VLF), low frequency (LF), high frequency (HF), total power (TP), LF/HF ratio, heart rate, and HRV index, will be analysed at baseline and 5 and 13 weeks after baseline. This test will be performed at DongGuk University Ilsan Oriental Hospital only.

BMI (body mass index) is the most commonly used indicator of obesity and is determined from height and weight. In this trial, BMI will be assessed at baseline and 5 and 13 weeks after baseline.

Laboratory examination will be performed for the lipid profile, hs-CRP, fasting blood glucose, and uric acid. The lipid profile measures total cholesterol, HDL cholesterol, LDL cholesterol, and triglyceride levels. Blood samples will be collected after overnight fasting at baseline and at weeks 5 and 13. An overview of the outcome measurement time points is presented in Table 1.

**Table 1:** Schedule for treatment and outcome measurements

**Period**	**S**	**T**								**F**	
Visit	1	2	3	4	5	6	7	8	9	10	11
Week		1		2		3		4		5	13
Informed consent	●										
Inclusion/exclusion criteria	●										
Random allocation		●									
Auricular acupuncture treatment		○	○	○	○	○	○	○	○		
BP before and after auricular acupuncture treatment		○	○	○	○	○	○	○	○		
24-h ABPM	●					●				●	●
Laboratory examination		●								●	●
BMI	●					●				●	●
EQ-5D		●								●	●
Heart rate variability	●									●	●
Safety assessment		●	○	○	○	○	○	○	○	●	●

*S* Screening period, *T* treatment period, *F* follow-up period, *BP* blood pressure, *ABPM* ambulatory blood pressure monitoring, *BMI* body mass index, *EQ-5D* EuroQOL-5D.

●: Both the auricular acupuncture group and the usual care group.

○: Only the auricular acupuncture group.

### Sample size

There is a growing body of research evaluating the effect of acupuncture for various diseases, including hypertension. However, there is still a lack of studies evaluating the effect of auricular acupuncture on prehypertension or stage 1 hypertension. There is no previous study on which to base the sample size calculation. The current study is designed as a pilot study to determine initial data for the primary outcome measure to perform a sample size calculation for a large-scale randomised controlled trial. Each group will include 30 participants, which is the minimum sample size for evaluating the effect of auricular acupuncture. More than 80% of all eligible patients can be recruited, and more than 80% of all randomised patients have to complete the scheduled treatments, assessments, and follow-up [29-31].

### Statistical analysis

All analyses will be performed with SAS (version 9.1.3; SAS Institute, Inc., Cary, NC, USA) by a statistician blinded to the allocation of groups. Statistical analysis will be undertaken on the intent-to-treat (ITT) basis with a 95% confidence interval using multiple imputations. The ITT analysis will include all patients who are randomised [32], and the per-protocol (PP) analysis will include patients who complete the study and do not have major protocol violations [33]. All analyses will be based on the ITT population, and the result of the ITT analysis will be compared with the PP analysis to assess the sensitivity.

Analysis of covariance (ANCOVA) with the baseline score as a covariate for the whole subject pool will be used to assess differences in treatment outcomes between the two groups at each of these time points as a main analysis. The results for the applicable stratified groups with centre, age, and sex as stratified variables will be assessed as additional analyses. The repeated measures analysis of variance will be performed for the different time point assessments.

### Adverse events

Any expected or unexpected adverse events will be reported by the participants and practitioners at every visit and followed up to completion. The adverse events known to be related to auricular acupuncture treatment include local bleeding or pain at the needle insertion points, local redness, itching, and dizziness during treatment and the follow-up period [17]. If any adverse events occur, the patient will be provided appropriate treatment. Serious adverse events will be reported to the principal investigator immediately, and the patient will be withdrawn from the clinical trial; details, including the date of occurrence, lost time, measures taken related to the treatment, causal relationship with the treatment, and treatment of the adverse event, will be recorded.

### Ethics

This research protocol has been reviewed and approved by the institutional review boards of each trial centre (Daejeon University Hospital [djomc-72] and DongGuk University Ilsan Oriental Hospital [2011–15]). Written informed consent will be obtained from all study participants prior to enrolment into the study.

## Discussion

The initial management of hypertension begins with a diagnosis based on several blood pressure measurements made in the clinic. Ambulatory blood pressure (ABP) is a more accurate measure of BP compared with clinical BP and shows better correlation with cardiovascular outcomes and target organ damage [34-36]. Some studies also suggest that adopting ABP monitoring as the reference standard for the diagnosis of hypertension would lead to more appropriately targeted treatment, and this would be particularly important for patients near the threshold for diagnosis [37,38]. Therefore, we will use ABP as a primary outcome measurement to assess the effect of auricular acupuncture on prehypertension and stage 1 hypertension. The clinical blood pressure will be assessed as a secondary outcome measurement.

Blood pressure is a surrogate end point, and the relationship between blood pressure and the risk of morbid cardiovascular events is well established [39,40]. The present pilot study will provide data on the effectiveness of auricular acupuncture for reducing blood pressure and the duration of therapy through a follow-up period of 2 months after the completion of the treatment. An assessment of reductions in clinical outcomes, such as cardiovascular events, stroke, and coronary heart disease, and the prevention of progression from prehypertension to stage 1 hypertension will be needed in a future long-term clinical trial.

In addition to appropriate outcome measures, the use of an appropriate control group is a critical issue in designing a high-quality clinical trial. In prehypertension and mild hypertension, lifestyle modification is the first line and an essential step for lowering blood pressure before starting lifelong antihypertensive medication [2,4]. The purpose of this study is to clarify whether acupuncture is effective in controlling the blood pressure of patients with pre- and mild hypertension if implemented in real-world clinical conditions, and we have planned a pragmatic design using usual care as the control group. A pragmatic clinical trial can provide evidence focussed on the effectiveness of an additional treatment in a real-world setting rather than the efficacy of a treatment in an ideal experimental setting [41,42]. This pragmatic comparison of acupuncture with usual care has some limitations. First, not blinding the participants to the intervention and potential placebo effects is related to overestimation of intervention effects. Second, the inevitable difference in number of visits and clinic BP measurements between the two groups can bias the result. To minimise the confounding effects, any contact and conversation not involved in acupuncture treatment between researchers and patients are prohibited, and there is no mention of or advice on BP and lifestyle. The number of primary outcome measurements is identical in the two groups. Baseline characteristics, including smoking, sodium intake, alcohol intake, and physical activity, will be obtained and assessed in both groups. Patients’ pre-treatment expectations will be measured using a modified question of the Treatment Expectancy Questionnaire, which is “How much do you feel auricular acupuncture therapy will help to lower your blood pressure?” This question will be scored on a 9-point numeric rating scale (1 = not at all; 9 = very much) [43-47]. The study results will be interpreted cautiously.

In conclusion, this pilot multicentre randomised controlled trial will provide important clinical evidence for the effect of auricular acupuncture on blood pressure in patients with prehypertension and stage 1 hypertension compared with usual care.

## Trial status

The trial is currently in the recruitment phase, and 54 patients were recruited.

## Abbreviations

SBP: Systolic blood pressure; DBP: Diastolic blood pressure; JNC7: Seventh Report of the Joint National Committee on Prevention, Detection, Evaluation and Treatment of High Blood Pressure; BP: Blood pressure; OTC: Over-the-counter; ABPM: Ambulatory blood pressure monitoring; BMI: Body mass index; EQ-5D: EuroQOL-5D; HRV: Heart rate variability; SDNN: Standard deviation of all of the normal-to-normal intervals; RMSSD: Square root of the mean squared differences of successive normal-to-normal intervals; pNN50: Percentage of successive normal interbeat intervals greater than 50 ms; VLF: Very low frequency; LF: Low frequency; HF: High frequency; TP: Total power; hs-CRP: High-sensitivity C-reactive protein; HDL: High-density lipoprotein; LDL: Low-density lipoprotein; ITT: Intent-to-treat; PP: Per-protocol; ANCOVA: Analysis of covariance; ABP: Ambulatory blood pressure.

## Competing interests

The authors declare that they have no competing interests.

## Authors’ contributions

JHK drafted this manuscript. HJJ and THK made a substantial contribution to designing the study protocol. JEK and SL participated in the critical revision of the manuscript. KWK participated in the design of the statistical analysis. SYJ, ARK, HJP, KMS, HJJ and MSS participated in the design of the outcome measurements and assessing the outcomes. SDL and KEH helped to draft the manuscript. SMC had final responsibility for the decision to submit for publication. All of the authors read and approved the final manuscript.
